# Genetic Profiling in Diffuse Large B Cell Lymphoma – the promise and the challenge

**DOI:** 10.1016/j.modpat.2022.100007

**Published:** 2023-01-01

**Authors:** Natasha H. Cutmore, Joanna A. Krupka, Daniel J. Hodson

**Affiliations:** Wellcome-MRC Cambridge Stem Cell Institute and Department of Haematology, University of Cambridge, Cambridge, CB2 0AW, UK

## Abstract

Diffuse Large B Cell lymphoma (DLBCL) is the commonest non-Hodgkin lymphoma. Over the last two decades tremendous progress has been made in our understanding of the molecular pathogenesis of DLBCL. However, this biological understanding has not yet been translated into improved first-line therapy. A major barrier to the introduction of molecularly targeted therapy in DLBCL is the considerable molecular heterogeneity of this disease. Recent studies have tried to rationalise this heterogeneity by proposing new genetic subtypes of DLBCL. Whilst remarkable consensus exists over the broad nature of these genetic subtypes, important questions remain over precisely how, or even why, genetic subtyping might be incorporated into diagnostic laboratories. In this review we compare the findings of the major genetic subtyping studies and discuss the implications this may have for diagnostic pathology services and the management of DLBCL.

## Introduction

Diffuse large B cell lymphoma (DLBCL) is the most common adult lymphoid malignancy, and has an estimated worldwide incidence of 150,000 new cases per year^1^. With currently available first-line therapy, around two thirds of patients are cured. However, relapsed or refractory patients have a poor survival and many will die from their disease ^2^. R-CHOP, the standard of care immunochemotherapy for first-line treatment of DLBCL was introduced more than twenty years ago ^3^. Since then, a series of randomised trials, together enrolling more than 12,000 patients, has tested multiple modifications to R-CHOP including the incorporation of promising molecularly targeted drugs. However, results have been disappointing and twenty years later R-CHOP remains standard of care for first-line therapy of DLBCL ^4–13^. In this review we highlight the considerable molecular heterogeneity of DLBCL and argue that our inability to resolve adequately this heterogeneity with current diagnostic techniques is a dominant reason for our lack of progress in DLBCL trials. Put simply, molecularly targeted drugs are unlikely ever to show benefit until they are deployed in a molecularly targeted fashion. Emerging genetic classification systems may provide the key to rationalising this molecular heterogeneity and allowing molecularly targeted drugs to be tested in molecularly stratified trials. In this review we discuss these genetic classification systems, considering both the promise and the challenge of their introduction to clinical practice.

## Summary of existing subclassification methods

As we contemplate the introduction of newer genetic classifications, it is informative to briefly review the recent history of molecular profiling in DLBCL and, with the benefit of hindsight, to consider lessons that might be learned. The concept that DLBCL comprised more than one molecular disease was driven initially by the development of technology for whole transcriptome profiling ^14,15^. The most accepted transcriptional classification has been the Cell of Origin (COO), proposed by Staudt and colleagues in 2000 ^14^. They used DNA microarrays to divide DLBCL into two groups with gene expression profiles resembling either normal germinal centre B-cells (GCB), or reminiscent of *in vitro* stimulated peripheral blood B-cells (ABC) ^14^. This classification provided some prognostic information, with ABC cases associated with an inferior response to R-CHOP ^16^. However, an arguably more important consequence of COO classification has been to provide a framework on which much of our current understanding of DLBCL biology has been built ^17^. When considering the value of any future classification system, it is essential to consider separately the significance of any prognostic value from the significance of the biological understanding it will add. Translation of the COO subclassification system into diagnostic laboratories proved challenging, mostly due to limited availability of transcriptional profiling technology outside the research setting. Surrogate immunohistochemical algorithms were developed instead, but these did not replicate the classification as accurately as transcriptional methods ^18–20^. Analogous technical challenges and the resulting pressure to develop (over)simplified proxies are exactly the problems we now face as we contemplate the role of new genetic classification systems^21^. The COO classification system was eventually incorporated into the WHO classification of DLBCL in the 2016 revision ^20^.

The same edition of the WHO classification saw the recognition of *MYC*, *BCL2* and *BCL6* rearrangements in high grade B cell lymphomas as a new disease entity, separate from DLBCL, colloquially referred to as double hit/ triple hit high grade B cell lymphomas (DH/TH-HGBCL) ^20^. This was intended to reflect the finding that *MYC* and *BLC2* rearrangements by fluorescence in situ hybridisation (FISH) conveyed a dismal prognosis in retrospective cohorts ^22,23^. Subsequent studies applied FISH more universally to newly diagnosed DLBCL and confirmed an inferior, although not dismal, outcome of *MYC* /*BCL2* double rearranged cases. Furthermore, recent studies suggest that negative outcomes may be restricted to rearrangements between *MYC* and immunoglobulin partner loci ^22,24–26^. Because of the poor response to R-CHOP, double hit lymphoma patients are often treated with intensified chemotherapy regimens ^27,28^. However, this is not an approach that has ever been validated in a prospective, randomised trial. Moreover, because of firmly entrenched opinions in both directions this is not an approach that can now be tested in a randomised clinical trial. The lessons here, as we contemplate new classification systems, are firstly that we should exercise caution when interpreting early, retrospective prognostic data; and secondly that the adoption of subtype-specific therapies should be based upon evidence from prospective trials.

MYC-driven DLBCL that overlap with those identified as double hit by FISH can also be identified by transcriptional profiling and two groups have proposed transcriptional signatures. These subgroups groups are termed Molecular High Grade (MHG) or DHSig respectively ^29,30^. Cases of DHSig in which *MYC* or *BCL2* rearrangements are not detected by FISH frequently carried cryptic rearrangements detectable by next generation sequencing. Almost all showed a GC origin according to the COO classification and, like Burkitt lymphoma, may originate from the highly proliferative dark zone of the germinal centre. Like DH-HGBCL, these dark-zone lymphomas share a poor response to standard R-CHOP, however, optimal therapy for these subgroups remains unknown.

## Genetic analysis of DLBCL

Over the last decade a series of genomic sequencing studies have served to confirm the validity of the ABC and GCB distinction ^31–35^. GCB cases were enriched for mutations in chromatin modifying genes, whereas ABC cases were enriched for mutations that activate the nuclear factor kappa B (NFKB) pathway, especially through activation of the B cell receptor (BCR) pathway ^36,37^. The general pattern of mutation in DLBCL is multiple driver mutations per patient (average 7-17) with around 150 recurrently mutated driver genes, including a long tail of genes mutated in small numbers of patients ^38^. Co-enrichment between mutated genes also began to emerge (e.g., *MYD88* and *CD79B*; *BCL2*, *CREBBP* and *EZH2*). This clustering of mutations became the basis for the newly proposed genetic classifiers.

## Genetic subtypes of DBCL

More recently, three large studies from Harvard^39^, the National Cancer Institute (NCI) ^40^ and the UK Haematological Malignancy Research Network (HMRN) ^41^, have converged independently onto very similar genetic subclassifications for DLBCL based on the profile of mutations caried by individual cases. Two of these have undergone further modification and one has been released as an online tool termed LymphGen, available for public use ^42,43^.

The Shipp group from Harvard applied whole exome and targeted sequencing approaches to profile somatic mutations, copy number alteration and structural variants ^39^. Their cohort consisted of 304 cases, including archived biopsy material and samples from the RICOVER60 trial ^44^. A final set of 158 genetic features were used for computational clustering, resulting in five genetic clusters with discrete genetic signatures. Briefly, the C1 subgroup was comprised of cases enriched for *BCL6* rearrangement and *NOTCH2* mutations. The C2 subgroup was associated with biallelic loss or mutation of *TP53* as well as widespread somatic copy number alterations. The C3 subgroup was enriched for *BCL2* rearrangement and mutation of *KMT2D*, *CREBBP* and *EZH2*. The C4 subgroup cases were demonstrated to have somatic mutations of histone linker and core histone genes, as well as *SGK1* and RAS/JAK/STAT pathway genes. The C5 subgroup was characterised by gains of 18q, and mutation of *MYD88* and *CD79B*. The Harvard classification system assigned 96% of cases to one of these subgroups. The remaining 4% with no detectable driver mutation were assigned to a default subgroup called C0.

Around the same time, the Staudt group from the National Cancer Institute (NCI) performed DNA copy number analysis, whole exome, transcriptome, and deep targeted sequencing on 574 fresh-frozen biopsy samples from a patient population enriched for ABC and unclassified DLBCL ^40^. Their clustering methods assigned just under 50% of cases to one of four subgroups, each named after the genes most commonly mutated within that group. The MCD group showed recurrent *MYD88* and *CD79B* mutations and were mostly ABC DLBCL cases (closely resembling the Harvard C5 subgroup). The EZB group contained cases with *EZH2* mutations and *BCL2* rearrangement, the majority of which were GCB DLBCLs (closely resembling the Harvard C3 subgroup). BN2 group cases were enriched for *BCL6* fusions and *NOTCH2* mutations (recapitulating the Harvard C1 subgroup) and contained the greatest proportion of unclassified COO cases. *NOTCH1* mutations, found mostly in ABC cases, were mutually exclusive with *NOTCH2* mutations and were assigned their own group, termed N1. The N1 subgroup was unique to the NCI study as no equivalent was identified in the Harvard publication ^39^. More than 50% of cases remained in the NCI study remained unclassified and the authors speculated that further subtypes remained to be discovered.

Shortly after, Staudt et al revisited their unclassified patient cohort and described two further subtypes with similarities to the Harvard C2 and C4 clusters. These were termed A53 - enriched for aneuploidy and *TP53* mutations; and ST2 – enriched for *SGK1* and *TET2* mutations ^43^. Additionally, they used transcriptional profiling to identify a MYC-driven (DHSig+) subgroup of poor risk cases from within the EZB group. They developed a probabilistic classifier termed LymphGen to assign individual patients to one of these seven genetic subtypes. The LymphGen classification also recognises “extended” subtypes, where patients had a high probability of belonging to more than one group. With the addition of two further “core” subtypes and extended subtypes, the LymphGen classification system assigned 63% of cases to a genetic subtype. LymphGen is available as an online tool for public use and is designed to accept full exome, structural variant and targeted sequencing data. Importantly, the LymphGen tool is designed to work with “imperfect” data, returning a classification probability based on whatever data is submitted.

An independent study from the UK Haematological Malignancy Research Network (HMRN) used formalin fixed, paraffin embedded (FFPE) samples from 928 cases of DLBCL and a targeted sequencing panel of 293 genes commonly mutated in haematological malignancies ^41^. Translocation and gene fusion data were not included in the final clustering strategy as this data was not available for all cases. Using a statistical modelling approach different to that used by the Shipp and Staudt groups, five genetic clusters were identified. These overlapped strongly with clusters identified by Harvard and NCI groups, validating the veracity of those classification systems.

**Figure 1:**
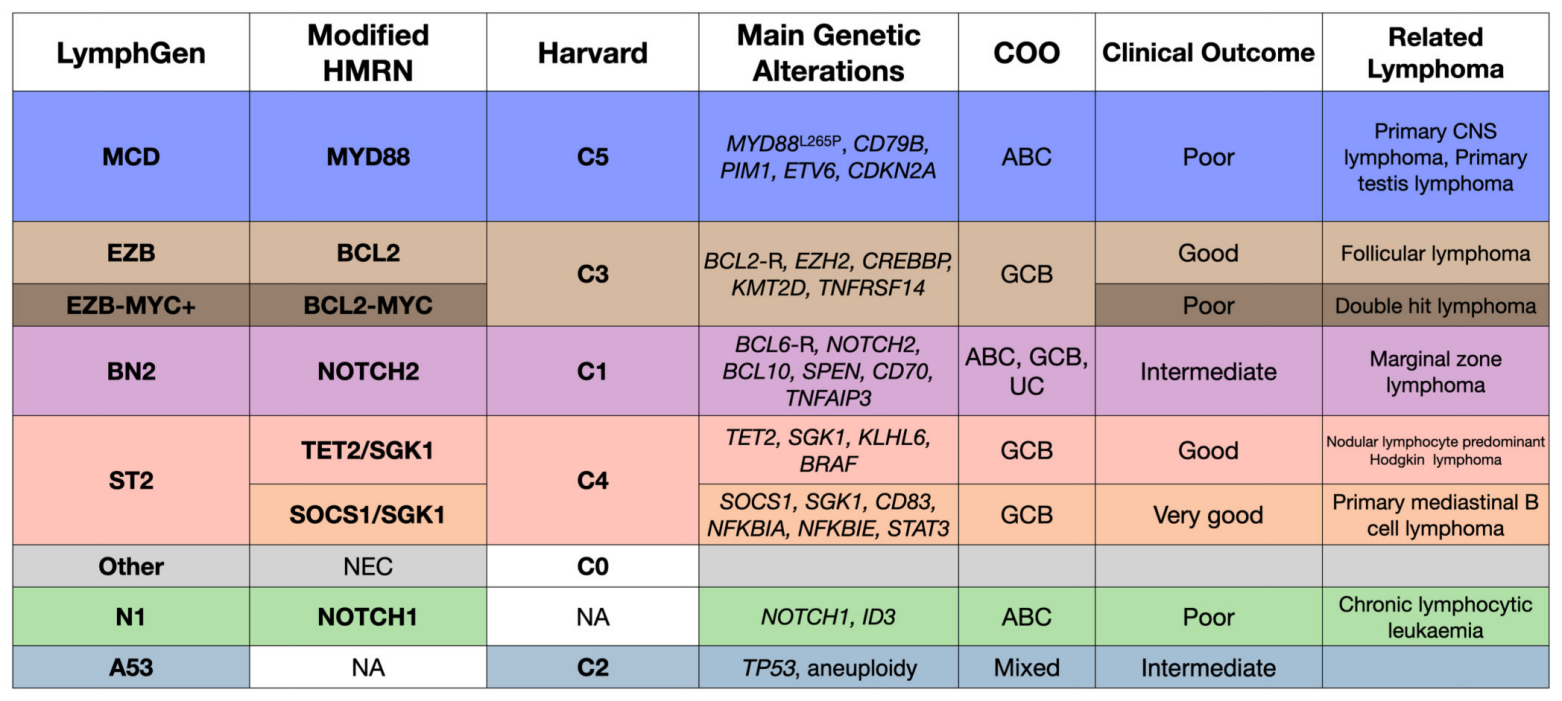
Overview of DLBCL genetic subtypes. Comparison of equivalent subtypes across the three main studies named according to LymphGen, Modified HMRN and Harvard nomenclature. *BCL2*-R and *BCL6*-R indicate rearrangement of these genetic loci.

**Figure 2:**
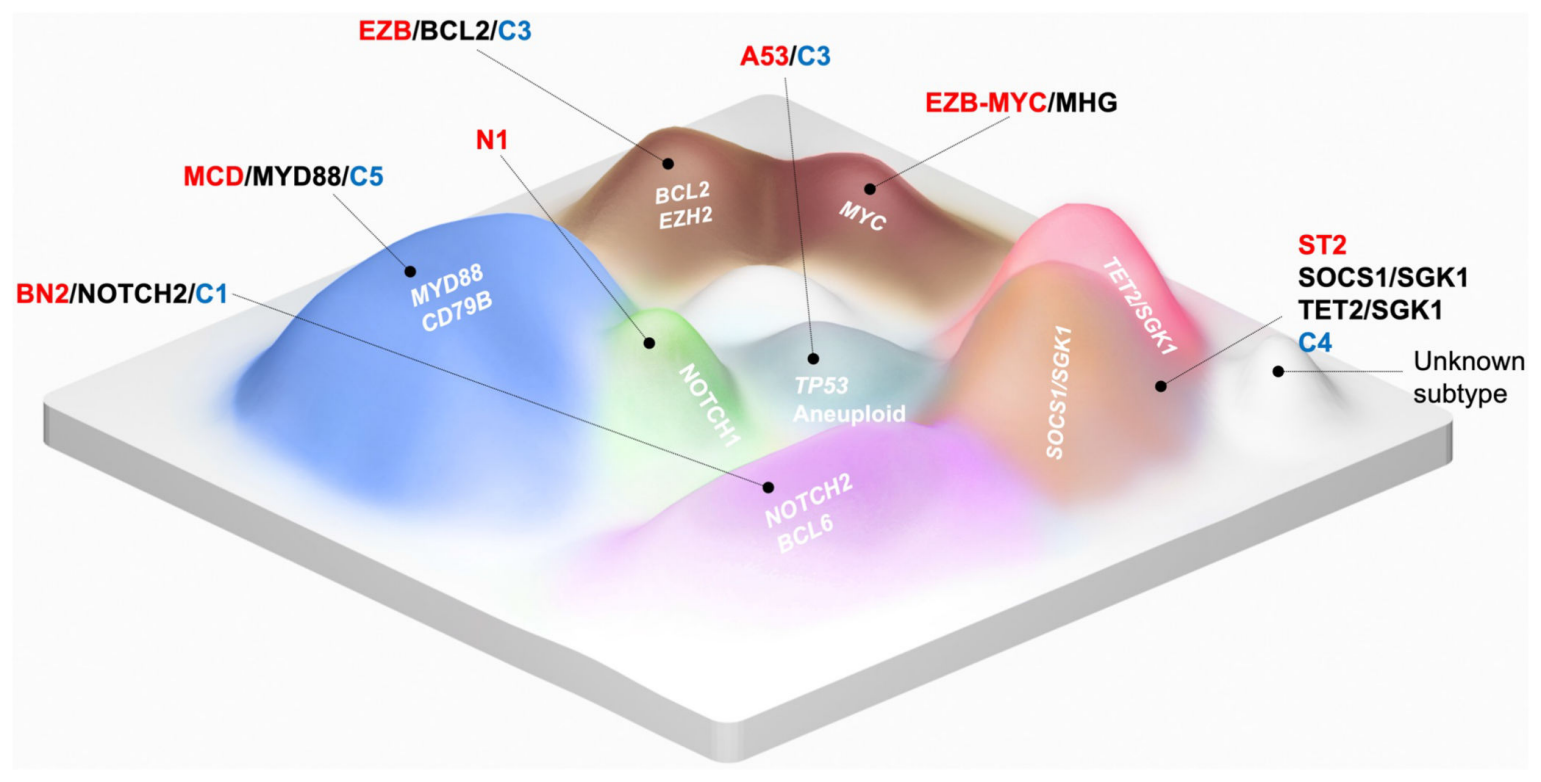
Topography of DLBCL molecular subtypes A conceptual representation of the genetic classification of DLBCL. Coloured hills depict known genetic subtypes. The gene mutations most enriched in each subtype are indicated in white. Each subtype is labelled with LymphGen (red), HMRN (black) and Harvard (blue) equivalent names. Whilst patients positioned on top of the coloured “hills” will be reproducibly classified by each classification system, patients positioned in the “valleys” may be unclassified or classified alternatively across different classification systems. Hills without colour correspond to unknown DLBCL subtypes which may emerge in the future from currently unclassified cases.

HMRN subgroups were named after the most enriched mutated genes. The “MYD88” subgroup had significant overlap with the MCD and C5 clusters; “BCL2” subgroup with the EZB and C3 clusters; “NOTCH2” subgroup with BN2 and C1 clusters. Perhaps due to the larger patient number in this study, the “SGK1” group (corresponding to the ST2/C4 clusters) was further divided into “SOCS1/SGK1” and “TET2/SGK1” subgroups. The clustering methods did not allow for the identification of a NOTCH1 subgroup due to the low number of *NOTCH1* mutant cases (1.7%), and the lack of copy number data meant that an A53/C2 group could not be identified. However, *NOTCH1* and *TP53* mutated cases were found to be enriched in the unclassified cases suggesting these subgroups might be resolved by alternative strategies. The HMRN classification system was subsequently modified to use truncating mutations in the *NOTCH1* PEST domain and a *MYC* mutational hotspot to identify “NOTCH1” and “BCL2-MYC” subtypes respectively ^42,45^. A comparison of genetic subgroups identified across each of these studies is shown in Figure 1 and conceptual “map” of DLBCL genetic subtype is shown in Figure 2.

## Relating genetic subtypes to existing pathological and clinical lymphoma subtypes

An intriguing observation made by each of the major studies was the association between the genetics of individual subgroups of DLBCL and that of other lymphoma entities, already known to us, including indolent lymphomas. The mutation profile of MCD/C5/MYD88, including mutations in the BCR, Toll like receptor (TLR) and NFKB pathways as well as immune evading mutations is highly reminiscent of those reported in extranodal lymphomas such as primary central nervous system lymphomas (PCNSL) and testicular lymphomas (PTL) ^46–49^. Indeed, almost all cases of PCNSL and PTL were classified into the analogous C5, MCD or MYD88 subgroups across all studies ^50^. The EZB/C3/BCL2 group shares an identical mutation pattern to that of follicular lymphoma (FL) ^35,51^. In the HMRN study, cases of transformed FL almost all clustered into this group. Moreover, in 27% of cases concurrent FL was identified on staging bone marrow biopsy ^41^. The mutation pattern of the BN2/C1/NOTCH2 group, including mutation of *NOTCH2*, *TNFAIP3*, and *BCL10* resembles that of marginal zone lymphomas (MZL) ^52,53^. It is possible these cases represent occult transformation from MZL, although direct evidence of this has not been observed. The ST2/C4 group is associated with activation of the JAK/STAT/ERK pathway and mutations in *SOCS1, DUSP2, STAT3* and *BRAF*, a profile shared with nodular lymphocyte predominant Hodgkin lymphoma ^54^. The SOCS1/SGK1 group identified in the HMRN study closely resembled primary mediastinal B-cell lymphoma (PMBCL) at both the transcriptional and genetic levels (mutations of *SOCS1, ITPKB, NFKBIE* and *CIITA*). This concurs with a previous report of PMBCL-like DLBCL identified from non-mediastinal sites ^55^. Finally, the N1/NOTCH1 group shows truncating mutations in the PEST degradation domain, leading to increased NOTCH1 activity ^56^. This mutation is frequently observed in chronic lymphocytic leukaemia cases and Richter’s transformation, although a biological link remains unproven ^57^. These observations suggest that genetic subtypes may evolve from, or at least share a common origin with, other lymphoma entities and shows how the molecular pathogenesis of DLBCL may straddle the boundaries of our current systems of lymphoma classification.

These associations with other histologically defined disease entities raise the question of whether genetic subtypes might be recognised by morphology or immunohistochemistry. However, whilst enrichment of certain morphological entities is seen within genetic subtypes, the bulk of cases are labelled as DLBCL NOS without any specific morphological or immunophenotypic clues to the underlying genetics. Although the histology was not reviewed in light of the genetic findings, this suggests that genetic subtypes cannot be predicted with sufficient accuracy using morphology or IHC. Given the obvious logistical advantage to the real time molecular subtyping of DLBCL this is likely to be an area of future research that might exploit findings from proteomic studies, perhaps incorporating advances in digital pathology and machine learning.

## Prognostic Implications of genetic subtypes

The genetic classifications divide DLBCL into subgroups that are distinct in their biology and pathogenesis. Whether they confer prognostic information is a question that should be considered separately. Across studies there are areas of agreement but also discordance, most likely related to the characteristics of patients enrolled in each study cohort. All studies agree that the ST2/C4/SGK1 group carried the most favourable 5-year overall survival (NCI= 84%, Harvard = 75%, HMRN = 80%). The poorest outcomes were observed in the N1/NOTCH1 group (NCI = 27%, modified HMRN = 40%) and the MYC positive subgroups of EZB (EZB-MYC^+^ 48% and BCL2-MYC 40% 5-year OS). The prognosis of the remaining groups showed variability across the different classification systems. A notable example is the MCD subgroup, which had an especially poor outcome in the NCI study (40% 5-year OS). In the HMRN study the MYD88 subgroup was also associated with inferior survival when considering all patients treated with “R-CHOP-like” regimens ^41^. However, the prognostic impact was much less evident when analysis was restricted exclusively to those treated with full dose R-CHOP. This highlights the importance of considering the clinical characteristics of patient cohorts recruited into individual studies and the need to recognise the differing biases introduced through use of pathology archives, clinical studies, and registry cases. Whilst the prognostic implication of genomic subtypes will become clear over time, it is important to recognise that the true value of genomic subtyping is not to inform on prognosis in response to R-CHOP, but rather to reveal distinct biological subtypes of disease that may respond differently to molecularly targeted therapies of the future.

## Molecular profiling reveals subtype-specific responses to targeted therapies

The identification of biologically discrete subtypes is the first step towards a precision medicine treatment approach in DLBCL. However, at the present time, genomic subtypes do not allow us to select different treatments for different patients. This is in part because previous trials have predominantly examined novel therapies in a blanket approach ^7,9,10,12,13,58–60^. Where molecular subtyping has been applied retrospectively, evidence of subtype-specific response to targeted therapy begins to emerge. The REMoDL-B study investigated the addition of Bortezomib to R-CHOP in a randomised trial. When considering all patients, no benefit was seen^59^. However, an unexpected trend towards improved survival was observed in the MYC-driven Molecular High Grade (MHG) subgroup identified by gene expression profiling^30^. This finding will need to be tested in future prospective studies but serves to illustrate how response to targeted therapies can only be resolved when DLBCL is considered by molecular subtype. A second example is the PHOENIX trial, which randomised patients with non-GCB DLBCL to R-CHOP plus either placebo or ibrutinib, an inhibitor of Bruton’s Tyrosine Kinase ^12^. Across all patients, no significant improvement was seen in the primary endpoint. However, a retrospective analysis, which classified patients into LymphGen genetic subtypes, revealed how ibrutinib was associated with significant survival improvement amongst younger patients classified into the MCD and N1 subtypes ^61^. Whilst the enhanced response of MCD subtype was predicted from preclinical data, the enhanced response of N1 DLBCL was not anticipated. Whilst the numbers were small, and conclusions require prospective validation, they demonstrate that genomic subtypes may begin to provide the granularity required to resolve clinical benefit of biologically targeted novel agents in future clinical trials. Finally, molecular profiling is not only relevant to targeted therapies, and colloquially termed “biology-agnostic” therapies are also influenced by tumour biology. Molecular profiling has revealed how CAR-T response is influenced by *TP53* status^62^ and how the activity of the CD79B antibody drug conjugate Polatuzumab is exerted preferentially in ABC transcriptional subtype of DLBCL^63^.

Overall, these findings support several conclusions; first, targeted therapies are unlikely to show efficacy when evaluated blanket-fashion across all DLBCL; second, when we apply adequate molecular profiling to clinical trials, subtype-specific responses are seen, although not always in the subtypes predicted from the biology; and finally, we argue that are no “biology-agnostic” therapies, only biology-agnostic trials.

## Ongoing evolution towards a final system of classification

Whilst current classification systems provide a promising starting point, several challenges must be addressed before they are considered ready for routine clinical use. The first is to reach consensus on a harmonised classification system. Whilst there is broad agreement over the nature of the genomic subtypes, there remain differences over precisely where to draw the boundaries (Figure 2). This is best exemplified by the widely different proportions of cases currently left unclassified; 4% in the Harvard classification, 27% in HMRN and 37% by LymphGen ^39,41,43^. It is likely that new subtypes will emerge from within these unclassified cases as greater numbers of patients are sequenced, and as they are subjected to greater levels of profiling complexity. Further layers of molecular data are likely to include novel non-coding mutations revealed by whole genome sequencing, microenvironment and host immune factors revealed by bulk transcriptome and single cell sequencing, and regulatory and expression changes revealed by epigenetic and proteomic profiling ^64–66^. This continued evolution and refinement makes the molecular classification of DLBCL a moving target, meaning careful consideration should be given to the profiling technology incorporated into future clinical trials. Whilst current genetic classifications can be reproduced with a relatively small, targeted gene panel, it seems essential that molecular profiling in clinical trials should include both full exome and full transcriptome profiling. This comprehensive molecular profiling is required to futureproof current trials. It provides the agility to apply the molecular classifications of the future, and to test hypotheses that emerge from discovery science without the need to repeat further molecular profiling as new questions arise. Finally, the development and refinement of future classification systems must progress hand in hand with the development of advanced preclinical model systems in which to decipher the biology and the targetable vulnerabilities of individual subtypes^67,68^.

## Integrating genetic profiling into routine diagnostic practice

Whilst the need for comprehensive molecular profiling of DLBCL in clinical trials is compelling, the requirement for genetic profiling in the routine diagnostic laboratory is less clear. Moreover, significant technical and logistical challenges currently preclude routine profiling in many centres. However, it is now only a matter of time before clinical trials establish subtype-specific therapies as standard of care in DLBCL. Therefore, leading diagnostic centres should act now to find ways to address these challenges to facilitate the integration of genetic profiling into existing diagnostic pathways. The speed and success of this process will depend critically upon the engagement of diagnostic pathologists.

Notable challenges include the availability of adequate tissue for molecular profiling. Despite the pleas of pathologists and lymphoma physicians, there is currently a widespread shift in practice from excisional biopsy towards needle core biopsies, meaning that residual material available for molecular analysis is often limiting. Formal excisional biopsy should be always encouraged for lymphoma diagnosis. However, where needle cores are taken, repeated cores must be embedded in separate blocks allowing one tumour-verified core to be reserved for molecular analysis. Where tissue is limiting, pathologists may need to order immunohistochemical studies judiciously to preserve material. Improved tissue handling protocols may include the collection of fresh biopsy material (with tumour content verified by flow cytometry) for molecular analysis, or the use of alternative fixatives that are less damaging to DNA. This may reduce formalin-associated sequencing artefact and increase the accuracy of variant calling, especially when whole exome or whole genome analyses are required. Alternatively, as the technology for analysis of cell free DNA continues to improve, it may be that plasma becomes a more practical source of tumour DNA to allow both mutation profiling and the inference of gene expression ^69,70^.

Diagnostic centres will need to make choices regarding the type of sequencing to perform. Outside of clinical trials and discovery science it seems likely that a focused panel provides the optimal balance of information versus sequencing costs. Further decisions including the sequencing platform and the bioinformatic pipeline for analysis will depend upon local factors and local expertise. At present, none of this is standardised across the lymphoma community and strategies for variant calling and driver annotation vary widely between the major academic sequencing studies. Whilst hotspot mutations such as *MYD88* L265P are reliably called as driver mutations, the reported frequencies of some non-hotspot mutations, such as those affecting *SOCS1* and *DTX1,* range from 0% to more than 15% across major DLBCL sequencing studies^38–41^. These findings emphasise how important it will be for the field to achieve a degree of standardisation and to develop robust systems for quality management. These challenges will not be overcome in academic studies and will require solutions built upon real world experience. Overcoming these challenges is an essential step towards effective precision medicine approaches in DLBCL.

## Conclusion

The diagnosis of DLBCL requires histological analysis of a tissue biopsy. We do not envisage that any molecular test will replace this requirement. However, genetic profiling now provides additional granularity to resolve biologically distinct subtypes of DLBCL. Despite the differing sequencing and computational approaches used, three major studies have now converged independently upon remarkably similar conclusions regarding the identity of these molecular subtypes. Whilst the molecular classification of DLBCL will continue to evolve, it seems likely that the genetic subgroups described above will represent the foundation to any future molecular classification. Genetic classification does not currently allow us to customise therapy for individual patients but provides a handle by which we can grasp the considerable molecular heterogeneity of DLBCL. After two decades of R-CHOP, emerging results now suggest that genetic subtyping may start to provide the granularity required to resolve benefit of molecularly targeted drugs in DLBCL trials. As such, comprehensive molecular profiling must now be a required feature of all DLBCL clinical trials. Outside of clinical trials, genetic profiling does not influence current management. However, it seems clear that future DLBCL therapies will ultimately be dictated by the biology of individual subtypes, in turn revealed by molecular profiling. Before such subtype-specific therapies can be implemented in the real world, considerable technical and logistical barriers must first be overcome. The early adoption of genetic profiling into routine diagnostic pathways and the active engagement of the diagnostic pathology community will allow us to overcome these challenges and to build the required infrastructure. Molecularly directed therapy is coming to DLBCL and diagnostic pathologists will be central to its implementation.

## Data Availability

Not applicable

## References

[1] Sehn LH, Salles G (2021). Diffuse Large B-Cell Lymphoma. New England Journal of Medicine.

[2] (HMRN), HMRN (2022). Survival statistics: Large B-cell lymphomas - Overall relative survival.

[3] Coiffier B, Lepage E, Briere J, Herbrecht R, Tilly H, Bouabdallah R (2002). CHOP chemotherapy plus rituximab compared with CHOP alone in elderly patients with diffuse large-B-cell lymphoma. N Engl J Med.

[4] Delarue R, Tilly H, Mounier N, Petrella T, Salles G, Thieblemont C (2013). Dose-dense rituximab-CHOP compared with standard rituximab-CHOP in elderly patients with diffuse large B-cell lymphoma (the LNH03-6B study): a randomised phase 3 trial. Lancet Oncol.

[5] Cunningham D, Hawkes EA, Jack A, Qian W, Smith P, Mouncey P (2013). Rituximab plus cyclophosphamide, doxorubicin, vincristine, and prednisolone in patients with newly diagnosed diffuse large B-cell non-Hodgkin lymphoma: a phase 3 comparison of dose intensification with 14-day versus 21-day cycles. Lancet.

[6] Schmitz N, Nickelsen M, Ziepert M, Haenel M, Borchmann P, Schmidt C (2012). Conventional chemotherapy (CHOEP-14) with rituximab or high-dose chemotherapy (MegaCHOEP) with rituximab for young, high-risk patients with aggressive B-cell lymphoma: an open-label, randomised, phase 3 trial (DSHNHL 2002-1). Lancet Oncol.

[7] Seymour JF, Pfreundschuh M, Trneny M, Sehn LH, Catalano J, Csinady E (2014). R-CHOP with or without bevacizumab in patients with previously untreated diffuse large B-cell lymphoma: final MAIN study outcomes. Haematologica.

[8] Jaeger U, Trneny M, Melzer H, Praxmarer M, Nawarawong W, Ben Yehuda D (2015). Rituximab maintenance for patients with aggressive B-cell lymphoma in first remission: results of the randomized NHL13 trial. Haematologica.

[9] Vitolo U, Trneny M, Belada D, Burke JM, Carella AM, Chua N (2017). Obinutuzumab or Rituximab Plus Cyclophosphamide, Doxorubicin, Vincristine, and Prednisone in Previously Untreated Diffuse Large B-Cell Lymphoma. J Clin Oncol.

[10] Witzig TE, Tobinai K, Rigacci L, Ikeda T, Vanazzi A, Hino M (2018). Adjuvant everolimus in high-risk diffuse large B-cell lymphoma: final results from the PILLAR-2 randomized phase III trial. Ann Oncol.

[11] Bartlett NL, Wilson WH, Jung SH, Hsi ED, Maurer MJ, Pederson LD (2019). Dose-Adjusted EPOCH-R Compared With R-CHOP as Frontline Therapy for Diffuse Large B-Cell Lymphoma: Clinical Outcomes of the Phase III Intergroup Trial Alliance/CALGB 50303. J Clin Oncol.

[12] Younes A, Sehn LH, Johnson P, Zinzani PL, Hong X, Zhu J (2019). Randomized Phase III Trial of Ibrutinib and Rituximab Plus Cyclophosphamide, Doxorubicin, Vincristine, and Prednisone in Non-Germinal Center B-Cell Diffuse Large B-Cell Lymphoma. J Clin Oncol.

[13] Nowakowski GS, Chiappella A, Gascoyne RD, Scott DW, Zhang Q, Jurczak W (2021). ROBUST: A Phase III Study of Lenalidomide Plus R-CHOP Versus Placebo Plus R-CHOP in Previously Untreated Patients With ABC-Type Diffuse Large B-Cell Lymphoma. J Clin Oncol.

[14] Alizadeh AA, Eisen MB, Davis RE, Ma C, Lossos IS, Rosenwald A (2000). Distinct types of diffuse large B-cell lymphoma identified by gene expression profiling. Nature.

[15] Monti S, Savage KJ, Kutok JL, Feuerhake F, Kurtin P, Mihm M (2005). Molecular profiling of diffuse large B-cell lymphoma identifies robust subtypes including one characterized by host inflammatory response. Blood.

[16] Lenz G, Wright G, Dave SS, Xiao W, Powell J, Zhao H (2008). Stromal gene signatures in large-B-cell lymphomas. N Engl J Med.

[17] Shaffer AL, Young RM, Staudt LM (2012). Pathogenesis of human B cell lymphomas. Annu Rev Immunol.

[18] Hans CP, Weisenburger DD, Greiner TC, Gascoyne RD, Delabie J, Ott G (2004). Confirmation of the molecular classification of diffuse large B-cell lymphoma by immunohistochemistry using a tissue microarray. Blood.

[19] Meyer PN, Fu K, Greiner TC, Smith LM, Delabie J, Gascoyne RD (2011). Immunohistochemical methods for predicting cell of origin and survival in patients with diffuse large B-cell lymphoma treated with rituximab. J Clin Oncol.

[20] Swerdlow SH, Campo E, Pileri SA, Harris NL, Stein H, Siebert R (2016). The 2016 revision of the World Health Organization classification of lymphoid neoplasms. Blood.

[21] Hodson DJ (2021). Diffuse large B-cell lymphoma genetics - simplifying the subtyping. Br J Haematol.

[22] Johnson NA, Savage KJ, Ludkovski O, Ben-Neriah S, Woods R, Steidl C (2009). Lymphomas with concurrent BCL2 and MYC translocations: the critical factors associated with survival. Blood.

[23] Niitsu N, Okamoto M, Miura I, Hirano M (2009). Clinical features and prognosis of de novo diffuse large B-cell lymphoma with t(14;18) and 8q24/c-MYC translocations. Leukemia.

[24] Rosenwald A, Bens S, Advani R, Barrans S, Copie-Bergman C, Elsensohn MH (2019). Prognostic Significance of MYC Rearrangement and Translocation Partner in Diffuse Large B-Cell Lymphoma: A Study by the Lunenburg Lymphoma Biomarker Consortium. J Clin Oncol.

[25] Copie-Bergman C, Cuillière-Dartigues P, Baia M, Briere J, Delarue R, Canioni D (2015). MYC-IG rearrangements are negative predictors of survival in DLBCL patients treated with immunochemotherapy: a GELA/LYSA study. Blood.

[26] Pedersen M, Gang AO, Poulsen TS, Knudsen H, Lauritzen AF, Nielsen SL (2014). MYC translocation partner gene determines survival of patients with large B-cell lymphoma with MYC- or double-hit MYC/BCL2 translocations. Eur J Haematol.

[27] Petrich AM, Gandhi M, Jovanovic B, Castillo JJ, Rajguru S, Yang DT (2014). Impact of induction regimen and stem cell transplantation on outcomes in double-hit lymphoma: a multicenter retrospective analysis. Blood.

[28] Oki Y, Noorani M, Lin P, Davis RE, Neelapu SS, Ma L (2014). Double hit lymphoma: the MD Anderson Cancer Center clinical experience. Br J Haematol.

[29] Ennishi D, Jiang A, Boyle M, Collinge B, Grande BM, Ben-Neriah S (2019). Double-Hit Gene Expression Signature Defines a Distinct Subgroup of Germinal Center B-Cell-Like Diffuse Large B-Cell Lymphoma. J Clin Oncol.

[30] Sha C, Barrans S, Cucco F, Bentley MA, Care MA, Cummin T (2019). Molecular High-Grade B-Cell Lymphoma: Defining a Poor-Risk Group That Requires Different Approaches to Therapy. J Clin Oncol.

[31] Morin RD, Mendez-Lago M, Mungall AJ, Goya R, Mungall KL, Corbett RD (2011). Frequent mutation of histone-modifying genes in non-Hodgkin lymphoma. Nature.

[32] Pasqualucci L, Trifonov V, Fabbri G, Ma J, Rossi D, Chiarenza A (2011). Analysis of the coding genome of diffuse large B-cell lymphoma. Nat Genet.

[33] Lohr JG, Stojanov P, Lawrence MS, Auclair D, Chapuy B, Sougnez C (2012). Discovery and prioritization of somatic mutations in diffuse large B-cell lymphoma (DLBCL) by whole-exome sequencing. Proc Natl Acad Sci U S A.

[34] Zhang J, Grubor V, Love CL, Banerjee A, Richards KL, Mieczkowski PA (2013). Genetic heterogeneity of diffuse large B-cell lymphoma. Proc Natl Acad Sci U S A.

[35] Morin RD, Johnson NA, Severson TM, Mungall AJ, An J, Goya R (2010). Somatic mutations altering EZH2 (Tyr641) in follicular and diffuse large B-cell lymphomas of germinal-center origin. Nat Genet.

[36] Young RM, Staudt LM (2013). Targeting pathological B cell receptor signalling in lymphoid malignancies. Nat Rev Drug Discov.

[37] Jiang Y, Dominguez PM, Melnick AM (2016). The many layers of epigenetic dysfunction in B-cell lymphomas. Curr Opin Hematol.

[38] Reddy A, Zhang J, Davis NS, Moffitt AB, Love CL, Waldrop A (2017). Genetic and Functional Drivers of Diffuse Large B Cell Lymphoma. Cell.

[39] Chapuy B, Stewart C, Dunford AJ, Kim J, Kamburov A, Redd RA (2018). Molecular subtypes of diffuse large B cell lymphoma are associated with distinct pathogenic mechanisms and outcomes. Nat Med.

[40] Schmitz R, Wright GW, Huang DW, Johnson CA, Phelan JD, Wang JQ (2018). Genetics and Pathogenesis of Diffuse Large B-Cell Lymphoma. The New England journal of medicine.

[41] Lacy SE, Barrans SL, Beer PA, Painter D, Smith AG, Roman E (2020). Targeted sequencing in DLBCL, molecular subtypes, and outcomes: a Haematological Malignancy Research Network report. Blood.

[42] Runge HFP, Lacy S, Barrans S, Beer PA, Painter D, Smith A (2021). Application of the LymphGen classification tool to 928 clinically and genetically-characterised cases of diffuse large B cell lymphoma (DLBCL). Br J Haematol.

[43] Wright GW, Huang DW, Phelan JD, Coulibaly ZA, Roulland S, Young RM (2020). A Probabilistic Classification Tool for Genetic Subtypes of Diffuse Large B Cell Lymphoma with Therapeutic Implications. Cancer Cell.

[44] Pfreundschuh M, Schubert J, Ziepert M, Schmits R, Mohren M, Lengfelder E (2008). Six versus eight cycles of bi-weekly CHOP-14 with or without rituximab in elderly patients with aggressive CD20+ B-cell lymphomas: a randomised controlled trial (RICOVER-60). Lancet Oncol.

[45] Cucco F, Barrans S, Sha C, Clipson A, Crouch S, Dobson R (2020). Distinct genetic changes reveal evolutionary history and heterogeneous molecular grade of DLBCL with MYC/BCL2 double-hit. Leukemia.

[46] Chapuy B, Roemer MG, Stewart C, Tan Y, Abo RP, Zhang L (2016). Targetable genetic features of primary testicular and primary central nervous system lymphomas. Blood.

[47] Braggio E, Van Wier S, Ojha J, McPhail E, Asmann YW, Egan J (2015). Genome-Wide Analysis Uncovers Novel Recurrent Alterations in Primary Central Nervous System Lymphomas. Clin Cancer Res.

[48] Bruno A, Boisselier B, Labreche K, Marie Y, Polivka M, Jouvet A (2014). Mutational analysis of primary central nervous system lymphoma. Oncotarget.

[49] Nakamura T, Tateishi K, Niwa T, Matsushita Y, Tamura K, Kinoshita M (2016). Recurrent mutations of CD79B and MYD88 are the hallmark of primary central nervous system lymphomas. Neuropathol Appl Neurobiol.

[50] Morin RD, Arthur SE, Hodson DJ (2022). Molecular profiling in diffuse large B-cell lymphoma: why so many types of subtypes?. Br J Haematol.

[51] Okosun J, Bodor C, Wang J, Araf S, Yang CY, Pan C (2014). Integrated genomic analysis identifies recurrent mutations and evolution patterns driving the initiation and progression of follicular lymphoma. Nat Genet.

[52] Rossi D, Trifonov V, Fangazio M, Bruscaggin A, Rasi S, Spina V (2012). The coding genome of splenic marginal zone lymphoma: activation of NOTCH2 and other pathways regulating marginal zone development. J Exp Med.

[53] Spina V, Khiabanian H, Messina M, Monti S, Cascione L, Bruscaggin A (2016). The genetics of nodal marginal zone lymphoma. Blood.

[54] Hartmann S, Schuhmacher B, Rausch T, Fuller L, Doring C, Weniger M (2016). Highly recurrent mutations of SGK1, DUSP2 and JUNB in nodular lymphocyte predominant Hodgkin lymphoma. Leukemia.

[55] Yuan J, Wright G, Rosenwald A, Steidl C, Gascoyne RD, Connors JM (2015). Identification of Primary Mediastinal Large B-cell Lymphoma at Nonmediastinal Sites by Gene Expression Profiling. Am J Surg Pathol.

[56] Fabbri G, Rasi S, Rossi D, Trifonov V, Khiabanian H, Ma J (2011). Analysis of the chronic lymphocytic leukemia coding genome: role of NOTCH1 mutational activation. J Exp Med.

[57] Rossi D, Rasi S, Fabbri G, Spina V, Fangazio M, Forconi F (2012). Mutations of NOTCH1 are an independent predictor of survival in chronic lymphocytic leukemia. Blood.

[58] Crump M, Leppa S, Fayad L, Lee JJ, Di Rocco A, Ogura M (2016). Randomized, Double-Blind, Phase III Trial of Enzastaurin Versus Placebo in Patients Achieving Remission After First-Line Therapy for High-Risk Diffuse Large B-Cell Lymphoma. J Clin Oncol.

[59] Davies A, Cummin TE, Barrans S, Maishman T, Mamot C, Novak U (2019). Gene-expression profiling of bortezomib added to standard chemoimmunotherapy for diffuse large B-cell lymphoma (REMoDL-B): an open-label, randomised, phase 3 trial. Lancet Oncol.

[60] Tilly H, Morschhauser F, Sehn LH, Friedberg JW, Trneny M, Sharman JP (2022). Polatuzumab Vedotin in Previously Untreated Diffuse Large B-Cell Lymphoma. N Engl J Med.

[61] Wilson WH, Wright GW, Huang DW, Hodkinson B, Balasubramanian S, Fan Y (2021). Effect of ibrutinib with R-CHOP chemotherapy in genetic subtypes of DLBCL. Cancer Cell.

[62] Shouval R, Alarcon Tomas A, Fein JA, Flynn JR, Markovits E, Mayer S (2022). Impact of TP53 Genomic Alterations in Large B-Cell Lymphoma Treated With CD19-Chimeric Antigen Receptor T-Cell Therapy. J Clin Oncol.

[63] Tilly H, Morschhauser F, Sehn LH, Friedberg JW, Trněný M, Sharman JP (2022). Polatuzumab Vedotin in Previously Untreated Diffuse Large B-Cell Lymphoma. N Engl J Med.

[64] Arthur SE, Jiang A, Grande BM, Alcaide M, Cojocaru R, Rushton CK (2018). Genome-wide discovery of somatic regulatory variants in diffuse large B-cell lymphoma. Nat Commun.

[65] Steen CB, Luca BA, Esfahani MS, Azizi A, Sworder BJ, Nabet BY (2021). The landscape of tumor cell states and ecosystems in diffuse large B cell lymphoma. Cancer Cell.

[66] Kotlov N, Bagaev A, Revuelta MV, Phillip JM, Cacciapuoti MT, Antysheva Z (2021). Clinical and Biological Subtypes of B-cell Lymphoma Revealed by Microenvironmental Signatures. Cancer Discov.

[67] Caeser R, Di Re M, Krupka JA, Gao J, Lara-Chica M, Dias JML (2019). Genetic modification of primary human B cells to model high-grade lymphoma. Nat Commun.

[68] Caeser R, Gao J, Di Re M, Gong C, Hodson DJ (2021). Genetic manipulation and immortalized culture of ex vivo primary human germinal center B cells. Nat Protoc.

[69] Scherer F, Kurtz DM, Newman AM, Stehr H, Craig AF, Esfahani MS (2016). Distinct biological subtypes and patterns of genome evolution in lymphoma revealed by circulating tumor DNA. Sci Transl Med.

[70] Esfahani MS, Hamilton EG, Mehrmohamadi M, Nabet BY, Alig SK, King DA (2022). Inferring gene expression from cell-free DNA fragmentation profiles. Nat Biotechnol.

